# Nucleases and Their Inhibitors: Exploring Biological Roles, Industrial Applications, and Challenges in Heterologous Expression

**DOI:** 10.1002/biot.70303

**Published:** 2026-08-27

**Authors:** Wian Vermeulen, Anton Du Preez van Staden, Leon M. T. Dicks

**Affiliations:** ^1^ Department of Microbiology Stellenbosch University Stellenbosch South Africa

**Keywords:** biochemistry, biology, cell biology, DNA damage, DNA mismatch repair, DNA repair, heterologous expression, homologous recombination, nuclease, nucleotide excision repair

## Abstract

Nucleases hydrolyze phosphodiester bonds and participate in numerous cellular and metabolic processes. Intracellular nucleases repair nonfunctional or damaged DNA using DNA base excision repair (BER), mismatch repair (MMR), and homologous recombination (HR). Apoptotic nucleases systematically degrade cellular DNA during programmed cell death (PCD). Non‐apoptotic nucleases support DNA repair and replication. Small noncoding RNAs (sncRNAs) degrade the RNA of viral particles. Extracellular and membrane‐associated nucleases replenish nucleotides, especially in biofilms where cells rely on additional carbon, phosphorus, and energy. Restriction endonucleases (REs) are indispensable in recombinant DNA technology. Some genetic disorders and cancers have been treated by changing the genetic code of host cells using the CRISPR (clustered regularly interspaced short palindromic repeats)‐Cas (CRISPR‐associated proteins) system. Nucleases are also used in vaccine development. Heterologous expression of nucleases remains challenging, largely due to cytotoxicity and product instability. Some successes have been reported using the T7 promoter‐based system. However, due to the formation of inclusion bodies (IBs), the nucleases were insoluble and of low activity. Refolding misfolded nucleases from IBs, tight control (sequestration) of periplasmic secretion, and coexpression with natural inhibitor proteins increased yield, purity, and biological activity. This review addresses the significance of nucleases, heterologous expression, gene regulation, activity inhibition, and product yield.

## Graphical Abstract and Lay Summary

Intracellular nucleases, depicted as blue circles in a nucleosome, hydrolyze phosphodiester bonds, repair damaged DNA using DNA base excision repair (BER), mismatch repair (MMR), and homologous recombination (HR), and are involved in DNA replication. Apoptotic nucleases systematically degrade cellular DNA during programmed cell death (PCD). Small noncoding RNAs (sncRNAs) degrade the RNA of viral particles. Non‐apoptotic nucleases support DNA repair and replication. Extracellular and membrane‐associated nucleases (yellow circles) replenish nucleotides. Restriction endonucleases (REs) cut DNA. Created in BioRender. Dicks, L. (2026) https://BioRender.com/xehscqe.

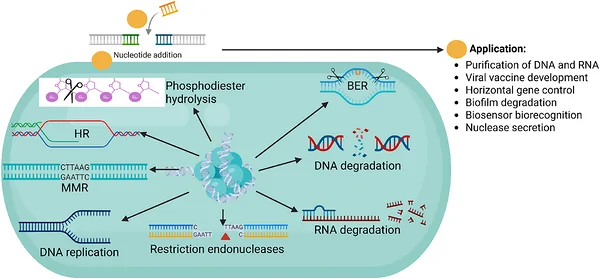

## 1 Introduction

Nucleases, or nucleolytic enzymes, are integral to a cell's survival and adaptation, participating in critical cellular processes [1]. Nucleases catalyze the cleavage of DNA or RNA by breaking the phosphodiester bonds between neighboring backbone residues [1]. Some nucleases display a preference for DNA or RNA, whereas others digest both and are known as non‐specific nucleases. Nucleases are used in gene manipulation, DNA replication, proofreading, topoisomerization, site‐specific recombination, transport and repair, RNA processing and maturation, apoptosis, and host cell defense [1]. They are grouped based on multiple classification criteria, including metal‐ion dependence and catalytic mechanism, each with separate subclassifications. Certain nucleases, such as restriction enzymes, digest specific nucleotide sequences and are broadly classified as endonucleases or exonucleases. Some nucleolytic enzymes act in a 5′ to 3′ direction, whereas others act in the opposite direction. Great diversity is also evident in the metal‐cation dependence of nucleases. Some nucleases are independent of metal cations, while others require the presence of one or two metal‐cation cofactors. In rare cases, three metal cations are required, such as nuclease P1 (*Penicillium citrinum*) and Endo IV (*Escherichia coli*), which require three Zn^2+^ ions. Moreover, the two most common metal‐cation cofactors required are Mg^2+^ and Ca^2+^; however, considerable diversity is observed here as well [2].

The human homolog of the apurinic/apyrimidinic (AP) nuclease, APE1 (also known as APX, APE, REF1, and HAP1), encoded by the APEX1 gene, is a multifunctional protein primarily recognized as the major enzyme in the DNA base excision repair (BER) pathway. APE1 repairs apurinic and apyrimidinic sites (3′‐5′‐exonuclease activity) damaged by radiation, oxidation, and chemicals [3]. The BER mechanism prevents mutagenesis, cytotoxicity, and cell death. Bifunctional nuclease (BFN1) degrades DNA and RNA and plays a major role in programmed cell death (PCD), senescence, and the recycling of nucleotides and phosphates [4]. In the domestic silk moth (*Bombyx mori*), a nonspecific nuclease is involved in viral defense by degrading viral dsRNA from cypoviruses [5]. In bacteria, the clustered regularly interspaced short palindromic repeats (CRISPR)‐Cas (CRISPR‐associated proteins) system plays a vital role in detecting and degrading invading viral nucleic acids [6].

Extracellular nucleases of bacteria degrade nucleic acids to provide host cells with additional nucleotides and phosphates [7]. They also function as virulence factors in pathogenic microorganisms and inhibit competing microbial growth by acting as bactericides. The universal nuclease (NucA) of *Anabaena* sp. PCC 7120 is secreted and serves as a virulence factor by aiding the establishment or invasion of competing colonies. The nuclease EndA from *Streptococcus pneumoniae* converts dsDNA into ssDNA, enabling natural transformation of the virulent cells [8]. In non‐competent *S. pneumoniae*, EndA serves as a virulence factor by degrading deadly DNA‐scaffold traps produced by neutrophils. NucA (*Staphylococcus aureus*) has also been implicated in biofilm dispersal and bacterial spread during infection. During lung infection in mice, NucA plays a crucial role in destroying neutrophil extracellular traps (NETs), which are commonly associated with the entrapment and killing of pathogens [9].

The first nucleases studied were bacterial restriction enzymes, reviewed by Santa et al. [10] and An et al. [11]. Over time, nucleases evolved to protect bacteria against bacteriophage DNA that hijacks cellular processes to complex regulators of immune functions [12, 13]. Bacterial restriction endonucleases (REs) are classified into four categories, that is, Type I, which recognizes specific DNA sequences but cuts the DNA at random, variable distances apart [14], Type II, which cuts with precision at specific nucleotides [12], Type III, which requires ATP and cofactors, recognizes specific DNA sequences, and cleaves strands into short fragments [15], and Type IV that targets methylated or hydroxymethylated nucleotides [16]. Type I restriction‐modification (R‐M) systems are large, hetero‐oligomeric complexes composed of an HsdR (host‐specificity DNA restriction) subunit that acts as a RE, trimers of HsdM (host‐specificity DNA modification) subunits that serve as methyltransferases, and an HsdS (host‐specific determinant specificity) subunit that guides HsdM to specific DNA sequences. HsdM and HsdS are required to form an active methyltransferase (MTase) [17].

The CRISPR‐Cas system, often referred to as a defense system or “immune system with memory,” allows bacteria to recognize foreign DNA (e.g., bacteriophage DNA). CRISPR‐Cas maintains a record of all “acquired” DNA sequences in the bacterial genome and can be compared to the human immune system, which relies on memory cells, such as B and T cells, to “remind” the body of previous infections. Cas9 nuclease is a powerful tool for genome editing and has opened new doors in medicine, including the editing of defective genes and the treatment of genetic disorders [18, 19].

TOUCAN (therapeutic oligonucleotides activated by nucleases) is used to target and destroy pathogenic cells and may serve as a smart drug delivery platform. A short, chemically modified oligonucleotide sequence that incorporates a therapeutic nucleoside analog (the “drug cargo”) is resistant to nonspecific host nucleases but is degraded by a specific nuclease produced by the target organism (pathogen) or by a nuclease associated with a particular condition (e.g., cancer or inflammation). Upon nuclease‐mediated cleavage, the therapeutic nucleoside analog is released at the site of infection or inflammation and internalized by the target cells (pathogen or inflamed cells), leading to cell death. For further information, see the review by Borsa et al. [20].

Nucleases are often viewed as defensive, protecting the genome from invading genetic material. However, nucleases may also confer specific characteristics to microbiota, for example, the Holliday junction endonuclease *Hjc* from *Sulfolobus solfataricus*, which directs recombination junctions in sulfur‐rich, acidic springs [21]. Type III R‐M systems in the cyanobacterium *Prochlorococcus* function as innate immune (defense) systems by distinguishing its own DNA from invading (foreign) DNA, for examplr, bacteriophages [22]. DNase II in the soil‐dwelling amoeba *Dictyostelium discoideum* clears cellular debris after autophagy and apoptosis [23]. DNA mismatch repair (MMR) in the yeast *Saccharomyces cerevisiae* is resolved by the nuclease *Exo*1 [24]. The nematode *Caenorhabditis elegans* uses CPS‐6, an apoptotic nuclease, to prevent harmful immune activation [25], and the flatworm *Dugesia japonica* uses DNase II to regulate its immune system [26]. In higher life forms, such as the mollusk *Mytilus galloprovincialis*, immune responses are regulated by RNase T2, which acts as a chemokine [27], and in insects, such as *Drosophila melanogaster*, DNase II removes apoptotic DNA to prevent an upsurge in immune reactions [28]. In mammals, including humans, DNase I and RNase A maintain immune balance by degrading self‐DNA and viral RNA, thus preventing autoimmune responses and inflammatory reactions [29]. Humans have four DNase I genes, two DNase II genes, TREX1, TREX2, and DNase IL‐3 genes, plus several genes encoding RNases 1–8, RNase L, and RNase T2, as reviewed by Hernandez [30].

In a research facility, restriction nucleases are used to manipulate DNA and RNA or to clone DNA fragments into plasmids or genomes to generate recombinant (genetically modified) cells. Engineered nucleases such as CRISPR‐Cas9, zinc finger nucleases (ZFNs), and transcription activator‐like effector nucleases (TALENs) are used in genome editing, which forms the cornerstone of gene therapy and biotechnology processes, as reviewed by Reddy et al. [31]. Nucleases are also incorporated in the design of highly sensitive diagnostic platforms, such as SHERLOCK and DETECTR, to detect unique nucleic acids in pathogens, for example, SARS‐CoV‐2.

In this review, we discuss the significance of nucleases in vivo and in vitro, heterologous expression, gene regulation, inhibition, nuclease purification, genetic engineering, industrial applications, and strategies to increase product yield.

## 2 Classic Cellular/Extracellular Functions of Nucleases

### 2.1 DNA Repair

Unrepaired and misrepaired DNA leads to several medical implications [32, 33, 34, 35, 36]. Mutations of tumor repressor BRCA1 and BRCA2 genes are associated with breast and ovarian cancers [37], mutations in the WRN gene with premature aging (referred to as Werner syndrome), mutations in the BLM gene with Bloom syndrome, and mutations in genes encoding DNA repair proteins with Cockayne syndromes [38]. The accumulation of damaged DNA leads to aging and neurodegenerative disorders, such as Alzheimer's, Parkinson's, and Huntington's diseases [39], and immune deficiency, which is either primary (congenital) or secondary (acquired) [40]. Unresolved DNA damage can interfere with crucial metabolic enzymes involved in DNA replication, such as helicases and polymerases, leading to genomic instability [41, 42].

Damaged DNA is repaired by BER or nucleotide excision repair (NER). Small, non‐helix‐distorting base lesions are repaired by BER nucleases [43]. Apurinic and apyrimidinic (AP) sites that form from the removal of damaged bases are repaired by APE1 or the Mus (musculus) homolog, APEX, which degrades 1 to 10 bases flanking the damaged site, followed by DNA priming [44]. NER requires two endonuclease complexes, that is, XPF‐ERCC1 and XPG. XPF‐ERCC1 creates incisions at the 5′‐end and XPG at the 3′‐end of the damaged site [45]. This repairs bulky, helix‐distorting lesions caused by alkylating and cross‐linking chemical agents [44]. The gaps produced by NER and BER nucleases are filled by DNA polymerases. Deficiencies in NER and BER result in the accumulation of mutations and oxidative damage, leading to cancer, neurological and neurodegenerative diseases, increased susceptibility to oxidative stress, embryonic death, and accelerated tissue aging [46, 47, 48, 49, 50]. MMR removes replication errors and is rectified by exonuclease 1 (*EXO*1), although FANCD2‐associated nuclease 1 may also be used [51]. This process is initiated by MutL recognition of replication errors, which are excised by *EXO*1 in a 5′–3′ direction [52, 53, 54, 55, 56, 57]. The non‐homologous end joining (NHEJ) pathway relies on endo‐exonucleases such as Artemis, which degrade overhangs from double‐stranded breaks (DSBs) to create blunt ends [58, 59]. This facilitates the direct joining of two DNA ends without the requirement for a homologous template. However, this repair mechanism can introduce small errors. To compensate for these errors, the homologous recombination (HR) pathway, with sister chromatids as templates, is used. In HR, endonucleases such as MRE11 are required to produce short 3′ overhangs near the break, which are further digested by *EXO*1 nuclease [60, 61]. This results in long single‐stranded DNA (ssDNA) tails, which facilitate HR and DNA repair. As with NER and BER, nuclease deficiencies in the MMR, NHEJ, and HR pathways are directly associated with certain cancers, neurological disorders, inflammation, and immunodeficiency [62, 63, 64, 65, 66]. Direct reversal repair pathways do not require nucleases [44].

### 2.2 Apoptosis

Apoptosis is characterized by several physiological changes, including membrane blebbing, genomic DNA degradation, chromatin condensation, nuclear breakdown, assembly of apoptotic bodies, and cell death by phagocytosis [67]. Nucleases are essential elements of genomic DNA degradation and a hallmark of apoptosis. The overexpression of nuclease inhibitors and a lack of apoptotic nuclease expression have been associated with various cancers [68, 69, 70]. To date, two major apoptotic pathways have been described, namely the intrinsic or mitochondrial pathway, and the extrinsic or death‐receptor pathway [67, 71]. In both pathways, intrinsic or extrinsic stimuli activate procaspases, which in turn activate caspases, initiating apoptotic DNA cleavage by nucleases [72, 73]. Caspases 3 and 7 cleave inhibitors that regulate apoptotic nucleases [74, 75, 76]. The best‐studied apoptotic nuclease is DNA fragmentation factor (DFF40) and its inhibitor, DFF45 [73, 75]. Several homologues, such as the mouse caspase‐activated DNase (CAD) and its inhibitor (ICAD), *D. melanogaster* Drep2 and Drep4, nonspecific nuclease (nsNuclease), endonuclease G (EndoG), and their inhibitors (Drep3, Drep1, and EndoGI, respectively), have been described [77, 78, 79, 80]. Interestingly, EndoG is also involved in caspase‐independent apoptosis instigated by *Leptospira interrogans* infections [81]. In healthy cells, EndoG is localized in the mitochondrial intermembrane space, which separates it from the nuclear DNA. Compartmentalization acts as the primary mechanism of inhibition, preventing the enzyme from degrading the cell's genome [82]. In plants, leaf and stem senescence, abscission, and apoptosis in *Arabidopsis* spp. are genetically controlled processes tightly regulated by a complex network of internal and environmental signals. Bifunctional nuclease 1 (BFN1) is crucial to execute leaf and stem senescence, abscission, and apoptosis [4, 83]. The regulation of apoptosis is important for host cell defense, normal development, propagation by maintaining oocyte quality, and the removal of old and surplus cells [84, 85]. Dysregulation of apoptosis‐related nucleases can lead to various diseases, including cancer and autoimmune disorders, underscoring the necessity of precise nuclease activity in cellular homeostasis [86, 87]. The reutilization of nutrients is regulated by apoptotic nucleases [81, 82]. Apoptosis can restrict the spread of pathogens and tumor cells and prevent necrosis [84]. Nucleases play an important role in cell propagation and protect cells from disease outbreaks. Non‐apoptotic nucleases play essential roles in host cell defense to protect cells from invading pathogens and foreign nucleic acids.

### 2.3 Host Defence

Nucleases cleave nucleic acids and play a pivotal role in protecting host cells from foreign genetic invaders, such as viruses and bacteriophages. They degrade alien DNA or RNA before it hijacks the host cellular machinery. Viral RNA is degraded by cellular antiviral nucleases (e.g., RNase L) to prevent infection, or by viral‐encoded nucleases (e.g., SARS‐CoV‐2 nsp1) that degrade host‐cell RNA to hijack host protein synthesis [88, 89]. In the domestic silk moth (*Bombyx mori*), an nsNuclease is involved in viral defense by degrading invading viral dsRNA from cypoviruses. Similarly, in bacteria and archaea, CRISPR‐Cas and the MksBEFG complex in *Corynebacterium glutamicum* play vital roles in detecting and degrading invading viral nucleic acids. The MksBEFG complex consists of four proteins, that is, a structural maintenance of chromosome ATPase subunit (MksB), a kleisin‐interacting tandem winged‐helix element (MksE), a kleisin subunit (MksF) linking the MksB and MksE subunits, and the nuclease subunit (MksG). More than 48% of eubacteria and 95% of archaea possess one or more CRISPR‐Cas types, emphasizing the importance of this nuclease system as a defense mechanism [90].

Small noncoding RNAs (sncRNAs) degrade pathogen mRNA [91, 92]. The downregulation of host sncRNA biogenesis results in pathogen susceptibility. sncRNA thus acts as part of the innate immune system [93]. Two of the most well‐known examples of sncRNAs are PIWI‐protein‐associated RNAs (piRNAs) and microRNAs (miRNAs). Although the biogenesis of these small non‐coding RNA molecules differs, both require nucleases for processing and maturation. The Drosha ribonuclease complex (specifically, the RNase III enzyme Drosha, often linked to the DiGeorge critical region gene 8, DGCR8) cleaves the pre‐miRNA transcript within the nucleus, producing a shorter, approximately 70‐nucleotide stem‐loop structure referred to as the precursor miRNA (pre‐miRNA) [94]. The pre‐miRNA is converted into a duplex miRNA by the Dicer endoribonuclease and the Argonaute protein (e.g., AGO2 in humans), which forms the core of the RNA‐induced silencing complex (RISC) [95]. These miRNAs play important roles in virus detection and the immune system of various organisms [1, 96, 97, 98]. For example, knock‐out mutants of the Dicer endoribonuclease displayed enhanced sensitivity to pathogen infections [96]. Unlike miRNAs, piRNAs do not require the Dicer endoribonuclease. In contrast, additional nucleases, such as Zucchini endonuclease or PIWI proteins guided by complementary piRNAs, fulfill the processing and maturation steps of piRNA biogenesis [99, 100, 101]. After their maturation, piRNAs constitute an innate immune system response in various animals such as *M. musculus*, *D. melanogaster*, and *C. elegans* [99, 101, 102].

### 2.4 Extracellular Roles

Extracellularly, nucleases degrade nucleic acids to provide the host cell with nucleotides and phosphates [7]. Surface membrane‐associated nsNucleases are vital in obtaining nutrients, especially in aquatic environments or for organisms such as *Crithidia luciliae*, which cannot produce purines de novo [103, 104, 105, 106, 107, 108, 109, 110]. Similarly, nucleases degrade extracellular DNA to replenish their nucleotide pools and facilitate quick growth to high cell density and biofilm formation [110]. An extracellular nuclease of *Shewanella oneidensis* MR‐1 facilitates survival and propagation when DNA is the sole source of carbon, phosphorus, and energy [111]. This adaptation was likely a result of the metal‐reducing environments of *Shewanella* spp. containing low levels of soluble phosphates. Similarly, *Pseudomonas aeruginosa* secretes a DNase, facilitating the use of DNA as a nutrient source in cystic fibrosis patients [112]. This adaptation may be used to overcome plant and mammalian defence mechanisms against pathogens [113, 114, 115].

NETs are considered first‐line defence mechanisms used by mammalian cells [116]. Mammalian NETs consist largely of extracellular DNA, histones, and antimicrobial peptides, while plant‐like NETs comprise extracellular DNA, protein, and polysaccharides [113, 114, 115]. Bacteria, fungi, parasites, and viruses developed defense mechanisms, such as extracellular nucleases, to escape NETs. Examples are EndA, NucA, Dns and Xds, NucM, and Nuc produced by *S. pneumoniae*, *S. aureus*, *Vibrio cholerae*, *Moraxella catarrhalis*, and *Neisseria gonorrhoaea*, respectively [116, 117, 118, 119]. Biofilm formation increases the survival of NET‐targeted pathogens [117, 119]. Nucleases may even play a role in the transformation of genes encoding virulence and antibiotic resistance [8]. NucA of *Anabaena* sp. PCC 7120 serves as a virulence factor by aiding in the establishment or invasion of competing colonies [10]. EsaD of *S. aureus* inhibits the growth of competing bacteria [120].

## 3 Genetic Engineering

### 3.1 The Old

The discovery of “host‐controlled variation in bacterial viruses” in 1952 and 1953, explaining infection susceptibility and host range, prompted researchers to look beyond mutations and DNA methylation [121, 122, 123]. A decade later, type I and II REs were discovered [124]. However, these enzymes lacked the specificity required for host‐controlled variation. It was almost two decades after the discovery of host‐controlled variation, in 1970, that the mechanism behind this phenomenon was attributed to type II REs. The first described type II RE, *Hin*dIII, was isolated from *Haemophilus influenzae* serotype d [125]. Unlike type I and III REs, type II REs cleave within their short palindromic recognition sequences [126]. Furthermore, type II REs generate “sticky ends” or complementary overhangs, which are annealed with DNA ligase. Further development of recombinant DNA technology led to the first commercially available heterologous therapeutic, insulin [127]. More than 600 type II REs are commercially available [128, 129]. These enzymes are used to prepare recombinant plasmids, which facilitate access to cryptic genes and produce recombinant therapeutics and proteins for research purposes. However, despite their usefulness, type II REs cannot be used to genetically modify the genome of any given host due to their inability to form targeted DSBs, high cleavage frequency, and an overlap between DNA recognition and cleavage functions [6].

Advancements in molecular techniques have led to the development of programmable nucleases. These nucleases are more specific than type II REs, can be directed to cleave or replace certain sequences, and can therefore be employed in genomic engineering. A noteworthy example is ZFNs, which were also the first programmable nucleases. For further information on ZFNs, the reader is referred to the review by Chandrasegaran and Carroll [6]. In short, ZFNs were discovered through research on FokI, a type IIS RE, and the zinc finger motif. Unlike type II REs, FokI recognises a non‐palindromic DNA sequence and cleaves 9 to 13 nucleotides downstream of its recognition sequence [130]. Furthermore, FokI has separate DNA‐binding and non‐specific DNA cleavage protein domains, which allow replacement of the DNA‐binding domain with zinc finger proteins (ZFPs) [131, 132, 133]. These proteins recognise specific DNA sequences. In combination with phage display, multiple ZFPs were screened to identify their DNA‐binding sequences [15]. Subsequent protein engineering facilitated the coupling of multiple ZFPs with the nonspecific DNA‐cleavage domain of FokI to produce the first programmable nucleases. The benefits of programmable nucleases over REs allowed genome engineering of bacteria, yeast, plants, fish, mice germ lines, and bacteriophages [134, 135, 136, 137, 138, 139].

Decades of research on nucleases have enabled the development of REs for in vitro cloning and programmable nucleases for in vivo genome engineering. Further applications of these molecular tools enabled heterologous gene expression to characterize and access cryptic genes, produce commercial therapeutics, and facilitate fundamental research. However, the “old and improved” genetic engineering tools still had their drawbacks. Despite overcoming the inability of REs to facilitate in vivo genomic engineering, programmable nucleases suffered from laborious and time‐consuming preparation and multiple rounds of screening ZFPs for new targets. Therefore, an improved genomic engineering platform was required.

### 3.2 The New and Improved

The CRISPR‐Cas system, mentioned elsewhere, excises semi‐palindromic foreign sequences (termed “spacers”) and integrates them into bacterial and archaeal CRISPR loci [140]. This step serves to form a “memory” of previous viral infections and allows recognition thereof. Upon subsequent entry of the same viral nucleic acid, pre‐CRISPR‐RNAs are transcribed and matured into crRNA [141]. The crRNAs bind to trans‐activating crRNA (tracrRNA), enabling guided degradation of complementary foreign nucleic acids by Cas nucleases [6].

Realizing the potential of the CRISPR‐Cas system for genetic engineering, Charpentier and Doudna won the 2020 Nobel Prize in Chemistry for applying the CRISPR‐Cas system in genomic engineering [142, 143, 144]. The advantage of the system lies in its specificity. A further pivotal point was reached when Charpentier and Doudna found that the crRNA and tracrRNA need to be fused to form single‐guide RNA (sgRNA), thereby simplifying genetic engineering. Several different Cas nucleases from various organisms are used in genomic engineering, for example, Cas3, Cas6, Cas9, Cas12, and Cas14 [145, 146, 147, 148, 149]. This system generates DSBs at the target sequence specified by the sgRNA, which are repaired by host DNA repair pathways such as non‐homologous end joining [150]. This allows for insertions and deletions (collectively indels), which effectively change the genome. Alternatively, CRISPR‐Cas delivers exogenous donor DNA templates with flanking sequences that facilitate HR and specific editing of the target site. This approach has been used to model and study Alzheimer's disease in mice, for gene therapy, and to improve disease resistance and yields in crops [151, 152, 153, 154]. CRISPR‐Cas is a promising alternative to the treatment of genetic disorders and cancer [155, 156, 157, 158, 159, 160, 161]. Despite the success of CRISPR‐Cas genome editing, the system has drawbacks, including frequent indel byproducts, known as off‐target effects, that endanger successful genome editing [150]. Additionally, DSBs generated by CRISPR‐Cas nucleases are considered the most harmful form of DNA damage, potentially leading to genomic instability [44]. Alternative next‐generation editing technologies are required to overcome these challenges [150]. This may be provided by base and prime editing with nickases or mutant Cas nucleases that generate single‐stranded breaks [150, 162]. In the case of base editing, a deaminase is fused to the nickase, while prime editing consists of a nickase fused to a reverse transcriptase. Prime editing is a more versatile technique that allows indels or the replacement of multiple nucleotides as opposed to base editing, which can only change a small number of bases at a time [163] These technologies are used in similar applications to traditional CRISPR‐Cas systems, but they offer reduced off‐target effects and increased efficiency, while prime editing provides additional versatility [152, 156, 162, 163].

The discovery of REs, programmable nucleases, and Cas nucleases has paved the way for molecular biology, biotechnology, and gene therapy. However, nucleases are far more versatile than just their application in genetic engineering and have found numerous further applications in the purification of nucleic acids and heterologous proteins.

## 4 Heterologous Expression of Nucleases

The versatility and applicability of nucleases make efficient and robust heterologous overexpression of nucleases of great importance*. E. coli* is one of the most reliable and efficient hosts for heterologous expression, with nearly 50% of all heterologous proteins produced by *E. coli* [164] This expression host provides several advantages, such as its rapid doubling time, low culturing cost, fast protein production, availability of several genetic modification tools, generally regarded as safe (GRAS) status, and defined regulations for therapeutic protein production [165, 166, 167, 168]. Moreover, heterologously expressed proteins can represent more than 30% of *E. coli*’s total cellular protein [168]. Therefore, the majority of the discussion on heterologous nuclease expression will consider *E. coli* expression. However, reference is made to other expression hosts in the text and Table 1.

**TABLE 1:** Nuclease expression conditions, toxicity counter mechanism, and yield.

Nuclease	Origin	Toxicity counter mechanism	Expression parameters	Yield	Reference
Nonspecific nucleases					
NucA	*Anabaena* sp. strain PCC 7120	Inhibitor coexpression, short, IB^a^ expression, and high OD^a^	Induced at OD = 0.5 with 0.4 mM IPTG^a^, 28°C	90 mg/L	[204]
*sma*NucA	*S*. *marcescens*	IB expression and high OD	Heat shock induction at OD = 1 (28°C–42°C)	NA^a^	[260]
		IB expression	Heat shock induction (28°C–42°C)	NA	[261]
		Short, IB expression, and high OD	Heat shock induction at OD = 1 (28°C–42°C)	24 mg/L	[262]
SNase	*S. aureus*	Secretion	IPTG induction, 30°C	1 – 2 mg/L	[263]
		Secretion and high OD induction	Zn^2+^‐chelation induction at OD = 2, 37°C	115 mg/L	[264]
EndoG	Vertebrate and invertebrate spp.	Short, IB expression, and soluble expression	1 mM IPTG induction at OD 0.3–0.6, expressed for 3 h	Sol^a^: 116.56 µg/L and Insol^a^: 50.34 mg/L	[265]
EXOG	*Homo sapiens*			Sol: 203.52 µg/L and Insol: 61.48 mg/L	
CPS‐6	*C. elegans*			Sol: 273.48 µg/L and Insol: 40.49 mg/L	
Nuc1p	*S. cerevisiae*			Sol: 102.96 µg/L and Insol: 72.67 mg/L	
Nucyep	*Yersinia enterocolitica*	Short, IB expression, and high OD induction	IPTG or lactose induction at OD = 1.3, 1–6 h	25.5 mg/L	[266]
		Secretion and high OD induction	IPTG induction at OD = 0.8, 20.5 h, 32°C	NA	[267]
*Pin*Nuc	*Psychromonas ingrahamii*	Secretion and high OD induction	OD = 5–6, 30°C, up to 5 days	1 mg/L	[268]
*Pa*Nuc‐1	*Pantoea agglomerans*	Short, IB expression, and high OD induction	IPTG induction at OD = 40–45, 4 h, 37°C	NA	[269]
*Pa*Nuc‐2					
EsaD	*S. aureus*	Inhibitor coexpression and short expression	1 mM IPTG induction at OD = 0.5	NA	[120]
Ribonucleases					
RNase A	*Bos taurus*	Short IB expression	Induced with 1.0 mM IPTG, 37°C, 4 h	0.2 mg/L	[184]
		Periplasm localisation	Induction with 0.5 mM IPTG at OD = 0.5–0.6, overnight, 28°C	0.1 mg/L	[187]
		Short, IB expression	Heat‐shock induction at OD = 1.2 at 30°C, 25 min	2 mg/L	[185]
		Secretion and high OD induction	IPTG induction at OD = 1.0	45‐50 mg/L	[270]
RNase A	*B. taurus*	Secretion and high OD induction	MeOH induction at OD = 1, 72 h	1 mg/L	[271]
		Secretion	96 h expression	1 mg/L	[272]
		Secretion and high OD induction	Heat‐shock induction, 42°C, 14–20 h	100–300 mg/L	[183]
Barnase	*Bacillus amyloliquefaciens*	Inhibitor coexpression and secretion	NA	100 mg/L	[202]
		Inhibitor coexpression and secretion	Arabinose induction at 6 h, 37°C	225.9 mg/L	[179]
		Secretion and high OD induction	Constitutive, high OD = 4.6, 37°C	100–150 mg/L Highest: 500 mg/L	[207]
Deoxyribonucleases					
EBV^a^ alkaline DNase	Epstein–Barr virus	None	IPTG induction at an OD = 1.0, 6–7 h	30 µg/5 L (6 µg/L)	[173]
			Constitutive expression	230 µg/L	[273]
AsEndI	*Aliivibrio salmonicida*	Periplasm sequestration/secretion, and short expression	IPTG induction at an OD = 0.6, 7 h, 20°C	5.67 mg/L	[274]
Bp‐DNase I^a^	*B. taurus*	None	IPTG induction at OD = 0.4, 37°C	150 µg/L	[174]
Bp‐DNase I	*B. taurus*	None	IPTG induction at OD = 0.6–0.8, 37°C	2–3 mg/L	[175]
Colicin E9	*E. coli*	Inhibitor coexpression and high OD	IPTG induction at OD = 0.8	20–30 mg complex/L	[204]
CPAN/DFF40	Mammalian sp.	Inhibitor coexpression	Baculovirus transfection at 1.5 × 10^6^ cells, 25°C, 72 h	NA	[205]

^a^ Abbreviations: IB—inclusion body; OD—optical density (550 or 600 nm); IPTG—isopropyl‐β‐*D*‐1‐thiogalactopyrnoside; EPV—Epstein‐Barr virus; Bp‐DNase I—bovine pancreatic DNase I; NA—not applicable because the authors did not report; Sol—soluble; Insol—insoluble.

Despite the advantages of heterologous expression in *E. coli*, heterologous nuclease expression has faced several challenges. These include cytotoxicity, retarded growth rates, poor nuclease solubility, low yields, inefficient maturation and secretion, inefficient disulfide bridge formation, and metabolic burdens [169, 170, 171, 172, 173, 174, 175, 176, 177, 178, 179]. Most of these can be attributed to the degradation of the expression host's nucleic acids by the overexpressed nucleases. For example, early attempts to overexpress bovine pancreatic DNase I (bp‐DNase I) yielded no viable clones. In contrast, the expression of an inactive mutant of bp‐DNase I resulted in 20 mg/L of protein [174]. It was evident from this study and others, such as the expression of the nsNuclease from *Anabaena* sp. PCC 7120 that genetic regulation was the first hurdle [174, 180].

Seki et al. [3] reported no expression of APX nuclease in *E. coli* BW2001 cells, as well as aggregation following optimization. These have been overcome by genetic engineering, coexpression with soluble fusion partners or nuclease inhibitors, and secretion or periplasm targeting signals [169, 171, 181]. Shortle [170] expressed the SNase protein in *E. coli* strain HB10—as an appropriate starting point for optimization and expression studies due to its small size and lack of disulphide bridges. The author found that despite these two factors, the nuclease was only expressed at low levels since the yield obtained from heterologous overexpression was lower than that of the native producer. Moreover, the author speculated that the pre‐enzyme is not processed effectively. Despite this, it has been proven that expression optimization in *E. coli* can lead to the expression and correct folding of *Leishmania infantum* nuclease, a eukaryotic parasite [172].

Not only is the expression of a heterologous protein a metabolic burden on *E. coli*, but nucleases are also cytotoxic [173]. This can be attributed to their ability to degrade the heterologous host's nucleic acids, thereby making them lethal. Worral and Connolly [174] demonstrated the cytotoxicity of nucleases by comparing the expression of bovine pancreatic DNase I (bp‐DNaseI) and an H134Q active site mutant of this nuclease in *E. coli*. The authors found that the yield of bp‐DNase I was improved from 150 µg/L to 20 mg/L following mutagenesis. This can be attributed to the H134Q mutant only retaining 0.001% of the wild‐type bp‐DNase I activity [174]. Thus, it is evident that the nucleolytic activity of the wild‐type bp‐DNase I was responsible for low protein yield due to the associated lethality of heterologous host cells. However, the mutant protein was inefficient and therefore cannot be used or sold to industry or research laboratories. Therefore, cellular toxicity needs to be considered, and alternative approaches, including secretion, periplasmic localization, and the production of a temporarily inactive nuclease, can be employed to overcome the lethality of heterologous nucleases.

In literature, several steps have been taken to overcome this hurdle, with one of the most promising but underused methods being the coexpression of a nuclease with its natural inhibitor protein. Another interesting alternative to minimize the lethality of bp‐DNase I is the coexpression of antisense mRNA to prevent translation of the coding mRNA [175]. Following expression induction of the promoter regulating bp‐DNase I expression, the antisense mRNA‐producing promoter is essentially outcompeted, allowing the translation of bp‐DNase I mRNA. The same authors also expressed in a λDE3 lysogenic *E. coli* strain, which lacks T7 RNA polymerase required for transcription of the heterologous nuclease. Upon infection with *Escherichia* phage λCE6, carrying the T7 RNA polymerase gene, the nuclease was expressed. This enabled tight regulation of nuclease production and extended the viability of the heterologous host, resulting in denser cell cultures and, consequently, improved bp‐DNase I yields. Other attempts have focused on a combination of factors or a shorter expression period where induction occurs at much higher cell densities (OD_600_ = 1.0–1.5, 4–5 h expression) [176].

To overcome the inherent cytotoxicity of intracellularly expressed nucleases, the reported literature has also attempted to transport the nucleases to the periplasm. In a study by Takahara et al. [169], the SNase encoding gene was cloned into *E. coli* W620, which improved pre‐enzyme maturation and secretion compared with the pilot study by Shortle [170]. To achieve this, the OmpA signal peptide was fused to SNase, which targets the passenger protein to the periplasmic space. In the periplasm, the pre‐enzyme is matured and secreted, accounting for 60% of the total periplasmic proteins. Moreover, overexpression of SNase resulted in 12% of the total cellular protein being produced from pONF1, which is driven by the *lac* and *lpp* promoters. These authors also demonstrated the importance of a secretion signal since *E. coli* cells overexpressing SNase without the OmpA signal grew poorly. This can be attributed to the degradation of host nucleic acids, whereas the OmpA signal peptide clones allow periplasmic sequestration, thereby protecting host nucleic acids.

Cooke et al. [181] showed that OmpA‐NucB fusion protected *E. coli* JMN (previously JM107) from nucleic acid degradation. These authors, however, integrated *nucB* and *ompA* into the genome of *E. coli* strain JM107 and proved that following cell lysis, the host nucleic acids are degraded by the extracellular and periplasmic nuclease B. It was also demonstrated that no significant difference was observed between JM107 (plasmid expression of *ompA*‐*nucB*) and JMN (genome integration of *ompA‐nucB*) when comparing growth curves and specific growth rates. Nesbeth et al. [182] also reported on the cytotoxicity of staphylococcal NucA and therefore used the OmpA periplasm signal. The authors reported no negative effects on Fab fragment production or growth.

A US patent on the production of RNase A (from *Bos taurus*) was filed where the authors obtained > 100 mg/L [183]. This was achieved using the periplasmic phoA signal peptide. This patent showcased the intrinsic cytotoxicity of RNase A since previous reports got < 10 mg/L. Examples of this include 0.2 mg/L [184] using the lactose operator and T7 promoter, 2 mg/L [185] using a heat shock induction and inclusion body (IB) purification, and 4–8 mg/L [186] using a combination of T7 promoter and a fusion protein. The secretion or periplasmic sequestration of nucleases can successfully be employed as a strategy to counteract nuclease toxicity. However, it might not always work, as Tarragona‐Fiol et al. [187] reported a yield of 0.1 mg/L when RNase A was transported to the periplasm. Therefore, alternative methods should be studied, such as inhibitor coexpression.

**FIGURE 1:**
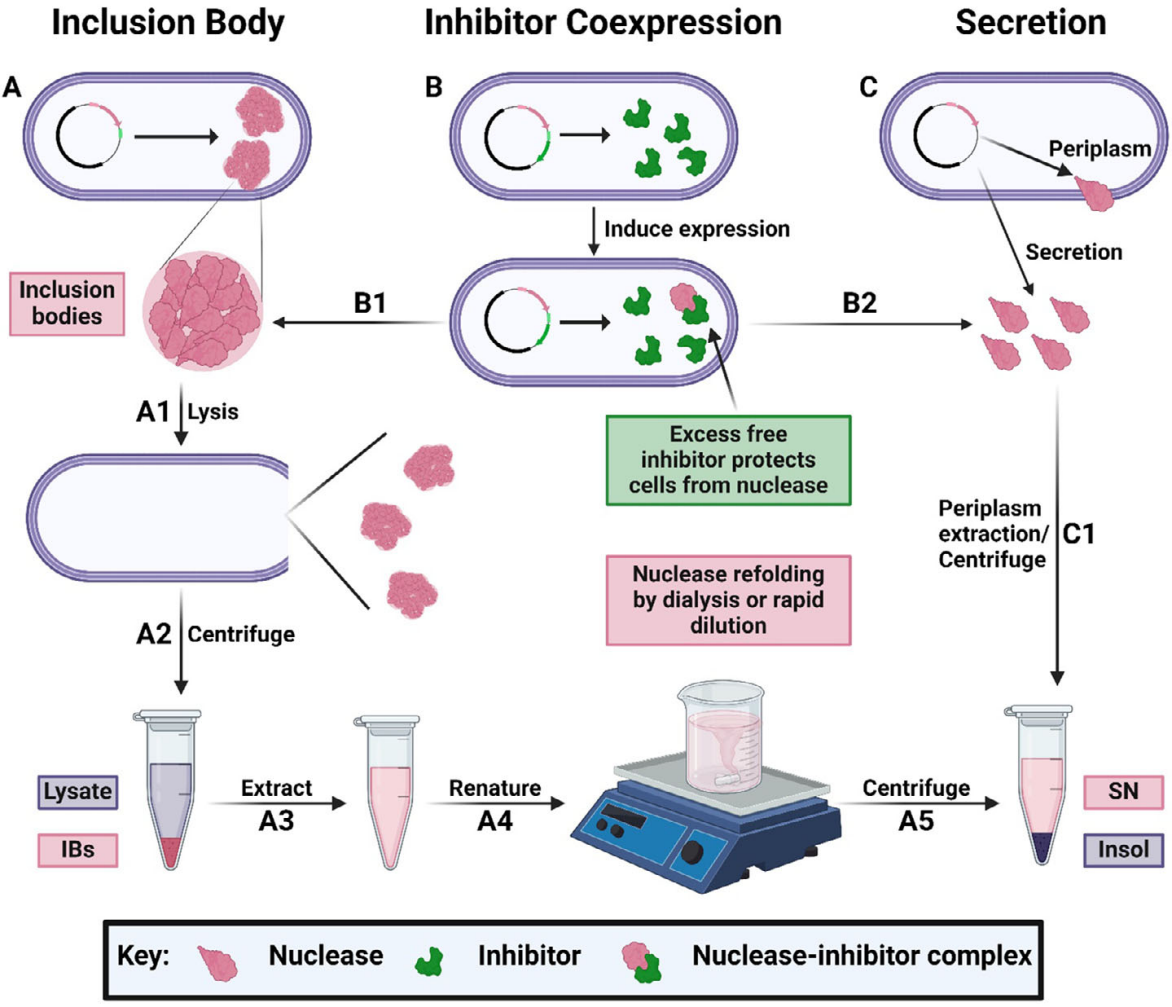
Heterologous expression and extraction of nucleases for purification. (A), (B), and (C) represent IB expression, inhibitor coexpression, and secretion, respectively. (A) Intracellular expression of nucleases results in IBs, which can be obtained by (A1) cell lysis and (A2) centrifugation. After centrifugation, nucleases in the pelleted IBs are (A3) extracted by denaturants, (A4) renatured for biological activity (*pink block*), and unfolded nuclease molecules or contaminating proteins are (A5) pelleted. (B) During inhibitor coexpression, an excess of nuclease inhibitor (*green block*) is required to ensure that the cytotoxicity of unintentionally expressed nucleases can be neutralized. After the expression of nucleases is induced, the extraction can either follow option (B1), which is similar to (A), or option (B2), which is like (C). (C) Secreted or periplasm‐sequestrated nucleases are acquired through (C1) centrifugation or periplasm extraction (see text). At the final steps of (A) and (C), the supernatant (SN in the figure) is retained and purified as discussed vide infra. IBs, inclusion bodies; Insol, insoluble fraction.

Heterologous overexpression by *E. coli* commonly results in unfolded, partially folded, and misfolded proteins that form inactive aggregates known as IBs [188, 189]. By forming IBs, *E. coli* protects itself from the cytotoxicity associated with the native conformation of nucleases (Figure 1A). Despite the inactivity of nucleases in IBs, their formation offers advantages, including ease of acquisition via centrifugation (Figure 1A, step A2), generally highly pure nuclease preparations (> 90%), and cost‐effective DSPs [190, 191]. Optimizing the extraction and renaturation of nucleases in IBs is therefore vital to ensure high yields of active nucleases. For example, initial expression of *Serratia marcescens* nsNucleases (commercialized as Benzonase) via secretion and periplasmic sequestration resulted in poor yields [192, 193].

It was later demonstrated that most of the nuclease is localized to IBs, thereby protecting *E. coli* from its cytotoxicity [176]. Similarly, the yield of RNase A was significantly increased when the nuclease was localized to IBs rather than produced via secretion or soluble intracellular expression (Table 1) [185, 186]. The low yields of secretion or soluble expression may be the result of proteolytic degradation, whereas RNase A in IBs is protected from proteases [185]. While this approach has improved the yields of several other nucleases (Table 1), the nucleases are inactive after IB solubilization (Figure 1A, step A3). This is due to the use of chemical denaturants, which are required to solubilize IBs. Therefore, the solubilized nuclease needs to be renatured in a refolding process, which has been successful in regaining the biological activity of nucleases.

During refolding (Figure 1A, step A4), physicochemical conditions that support protein stability and structural integrity are required. Furthermore, cofactors such as divalent cations, which are required for the structural maintenance and biological activity of nucleases, can be added [194, 195]. For example, various metal‐divalent cations have been found in the active site and in coordination bonds with nucleases such as Anabaena NucA, *Bacillus subtilis* NucB, and Benzonase [196, 197, 198]. Therefore, refolding has been used and validated for several nucleases. Refolding can be conducted by dialysis to slowly and gently change the physicochemical properties of the IB solubilisation buffer to those of the refolding buffer.

Care should be taken when the refolding approach is chosen, as refolding of certain nucleases can be challenging. While refolding of RNase A was achieved, had it not been for structural studies identifying the need for disulfide bridges for folding and biological activity, the nuclease would not have been able to undergo refolding [185]. In this case, the addition of oxidized and reduced glutathione facilitated disulfide bridge formation and enhanced biological activity. While no study has reported the inability of nucleases to be refolded, this process remains challenging, especially for complex proteins that require highly specific conditions [189]. As an alternative, secretion and periplasmic sequestration have also been employed to achieve successful nuclease expression by mitigating potential intracellular cytotoxicity.

As with the overexpression of nucleases targeted for secretion, unregulated overexpression of nuclease inhibitors can result in IB formation [199]. Regulating the expression of these inhibitors through weak promoters, such as the *trp* promoter, and using soluble fusion proteins has been used to overcome this hurdle [200, 201]. This approach not only resulted in soluble inhibitor expression but also improved its yield. Another successful approach entails low‐level constitutive expression of inhibitors such as barstar, the inhibitor of barnase, which yielded up to 1 g/L of barstar [202]. These approaches also set the foundation for inhibitor coexpression because, without coexpressing their inhibitors, nuclease cloning or induction of their expression results in cell death in heterologous hosts [202, 203, 204, 205, 206]. For this section, the two most well‐known inhibitor coexpression systems, *Anabaena* NucA and NuiA, and *B. amyloliquefaciens* barnase and barstar, will be used as prime examples.

The principle of coexpression is akin to that used by various organisms to protect themselves from their cytotoxic nucleases. For example, *Anabaena* sp. strain PCC 7120 always has low levels of NuiA, the inhibitor of NucA, in its cytosol through constitutive expression of NuiA. This mechanism ensures that unintentional expression of NucA in the cytosol is neutralized by the always‐available free NuiA. Similarly, Korn et al. [206] proved that the order of expression is important and that the inhibitor should be cloned upstream of the nuclease to allow its expression first. This approach resulted in significantly higher levels of NuiA, which facilitated the overexpression of NucA. However, low yields of NucA and NuiA were obtained. Interestingly, coexpression does not necessarily solve the insolubility of nucleases [202, 204, 207]. For example, the coproduction of NucA and NuiA resulted in IB formation, from which NucA had to be extracted and renatured (Figure 1B, option B1) [204]. Similarly, secretion combined with inhibitor coexpression is required to increase the yield of biologically active barnase (Figure 1B, option B2) [202, 203]. A potential disadvantage of inhibitor coexpression could be the separation of the nuclease and its inhibitor if expression in the cytosol is required or if the inhibitor is secreted or localized to IBs with the nuclease. However, in most cases, only the nuclease is localized to IBs or the extracellular environment, and separation is not required. Despite this, separation has been required for some nucleases and their inhibitors.

In the case of NucA and NuiA, urea is commonly used to solubilize IBs, which simultaneously facilitates the separation of the proteins by protein denaturation [204]. In this case, purification should be performed under denaturing conditions to ensure separation of the nuclease and its inhibitor. Alternatively, the nuclease domain of colicin E9 was separated from its inhibitor, Im9, by low pH (pH 2.5) and high salt concentration (0.5 M) [204]. While these approaches were successful, several buffer‐exchange steps were required to restore biological activity and structure. Specific proteases can also be used to facilitate the separation of a nuclease and its inhibitor. For example, the separation of caspase‐activated nuclease/DFF40 and its caspase‐sensitive inhibitor, DFF45, was performed by the proteolytic activity of caspase‐3 [205]. This protease plays a vital role in the regulation of apoptosis, and only upon appropriate apoptotic signals does it cleave DFF45 to produce mature DFF40, which will induce apoptosis by nucleic acid degradation [205, 208].

In conclusion, the heterologous overexpression of nucleases has traditionally been extremely challenging. However, through multiple innovations, nucleases could be overexpressed at high yields. These innovations include secretion, IB sequestration and refolding, and inhibitor coexpression. Importantly, all three approaches require rational genetic design to maximize yields. For secretion, it is vital to select a signal peptide that is efficiently processed and secreted by the heterologous host. In contrast, IB sequestration provides additional protection against the inherent cytotoxicity of nucleases, and inactive nuclease molecules can be refolded to their active form. Care should be taken during refolding, as all structural aspects of the biologically active nucleases should be considered when formulating an appropriate refolding buffer. While inhibitor coexpression remains an underused approach, it can benefit both secretion and IB sequestration strategies. Here, it is important to consider the genetic regulation of the inhibitor to ensure that it is always in excess compared to the nuclease.

## 5 The Application of Nucleases

### 5.1 In Purification

The role of nucleases in purification is often overlooked. Exogenous nucleases are used daily to purify gDNA, pDNA, RNA, and heterologous proteins from undesired nucleic acids [209]. Generally, RNases are used to degrade RNA during gDNA and pDNA preparations [209, 210, 211, 212]. Similarly, DNases are used to purify extracted RNA from contaminating DNA. The addition of exogenous nucleases reduces viscosity during protein purifications, thereby improving centrifugation, filtration, and chromatography. The process is, however, costly [213, 214, 215]. The coexpression of nucleases with the protein of interest may be a more viable approach [203, 215, 216, 217, 218]. Using staphylococcal nucleases (SNase) reduced the viscosity of cell lysates without affecting the production of biodegradable polyester [213]. Co‐expression of SNase is used in the production of viral vaccines by recombinant *E. coli* and mammalian cells [215, 219].

### 5.2 Viral Vaccine Development

Viral infections impose severe burdens on public health, livestock, and plants, necessitating innovative solutions to enhance vaccine development and availability. Recent examples include the coronavirus pandemic, the Orf virus in sheep and goats, and the cucumber mosaic virus in plants [220, 221, 222]. These challenges have driven the development of vaccines to ensure food security, agricultural sustainability, and human health. Developing live attenuated, inactivated, or recombinant viral or virus‐like particle vaccines must adhere to strict guidelines regarding production, purification, and testing [223]. According to FDA guidelines, the nucleic acid content of viral vaccines must be less than 100 pg/dose [224, 225]. High molecular weight nucleic acids contribute to sample viscosity, which interferes with polyethylene glycol precipitation, filtration, and liquid chromatography commonly used in viral vaccine preparations [226, 227, 228]. Removing nucleic acids from viral vaccines is thus a crucial step in production [190]. This was displayed in the production of benzonase endonuclease, frequently incorporated in detergent‐based lysis buffer to stabilize viruses and prepare simian adenovirus vaccines for clinical use [228, 229]. Nonspecific nucleases are used in the preparation of human rotavirus to prevent tangential flow during membrane filtration [230]. Treatment with nsNuclease shortened DSPs in the preparation of the VAQTA hepatitis A vaccine by increasing viral yield and vaccine purity [226]. Nucleases are also used in the preparation of vaccines against Ebola virus, human rotavirus, influenza, herpes simplex virus 1, and coronavirus [230, 231, 232, 233, 234].

Nucleases play an important role in bacterial ghost (BG) vaccines. BGs are cells devoid of cytoplasmic components, creating a “ghost” that cannot reproduce but retains its cell walls and surface antigens for immune recognition [235, 236]. They have been proposed as vaccine candidates, drug delivery vehicles, and immunomodulatory agents [236, 237, 238, 239, 240]. Traditionally, BGs were produced by intracellular expression of the PhiX174 phage endolysin, which lyses the host cell, leading to leakage of intracellular contents [241]. However, one major challenge remained: some BG cells retained viability, potentially leading to a disease outbreak or HGT. While the generation of nonviable BGs is extremely important to enable further applications, preventing HGT is a major concern. This concern not only affects BGs but also represents a major issue wherever recombinant DNA technology is used. The exchange of genetic material through HGT contributes to the spread of antibiotic resistance, the recurrence of human disease, and the disruption of ecosystems [242].

The concern of HGT in combination with the inability to generate completely nonviable BGs was one of the limiting factors in facilitating the translation of BGs from laboratories to clinical trials and human use [237]. This limitation was overcome by the coexpression of PhiX174 endolysin and an nsNuclease. Expression of SNase proved successful for this application by completely degrading nucleic acid, resulting in genetic inactivation [239, 243]. This removed the risk of potential HGT, potentially driving applications of BGs. Additionally, coexpression of an nsNuclease had no deleterious effects on BG preparation, yield, or quality [235, 243]. Further optimizations to nsNuclease expression have successfully been used to produce *E. coli* O92, avian pathogenic E. coli serotype O2, and *Vibrio anguillarum* BGs [244, 245]. All these preparations had high genetic inactivation and facilitated immune protection against *E. coli* O2 and *V. anguillarum*, with potential as vaccines for fish and birds [239, 245].

### 5.3 Horizontal Gene Control

Analogous to the role nsNucleases play in BG preparations, the controlled intracellular expression of nsNucleases has been employed to prevent HGT by other genetically modified expression hosts. For example, a conditional suicide system was genetically engineered into *S. cerevisiae* expressing *S. marcescens* nsNuclease (SmaNuc) under non‐laboratory conditions, such as glucose limitation [246]. This system, which induces intracellular SmaNuc expression upon glucose depletion, can combat possible environmental HGT by degrading all genetic material. A similar approach was successful in *E. coli*; however, it was limited to laboratory settings due to thermoinduction requirements [247].

While the environmental release of DNA and HGT is a major concern, the presence of cell‐free DNA (cfDNA) also poses several problems. Cell‐free DNA is found in NETs or biofilms during infection or as contaminants of pharmaceuticals [248, 249, 250]. Cancer progression, the development of lupus nephritis, inflammatory diseases, and persistent infections have been linked to the presence of cfDNA [251, 252, 253]. Therefore, alternative treatments and rapid and efficient detection of cfDNA are required. One such alternative for both treatment and detection is nucleases.

### 5.4 Biofilm Degradation

Persistent infections are commonly treated with *Lucilia sericata* larvae to debride necrotic tissue within the wounds, which have been found to produce an extracellular DNase [250]. This DNase facilitates the degradation of bacterial biofilms, which have DNA as a core component, and can therefore be used to treat chronic infections. Degradation of cfDNA and biofilm‐associated DNA is extremely important for good patient outcomes. For example, prolonged exposure to cfDNA, originating from NETs or biofilms, can lead to inflammation with several other inflammatory pathologies also linked with reduced DNase I activity [248, 253]. To investigate whether nsNucleases can resolve cfDNA‐induced inflammatory diseases, nsNucleases have been used to degrade NETs in mice. Indeed, these nsNucleases were able to ameliorate inflammation associated with ulcerative colitis in mice [253].

Although nsNucleases like SNase have shown promise in disrupting biofilm formation, their clinical use remains a challenging prospect due to potential limitations such as specificity and safety concerns. To circumvent the lack of specificity of nsNucleases, CRISPR‐Cas has been proposed to treat microbial infections. The CRISPR‐Cas system has shown promise in disrupting antibiotic‐resistance genes, treating bacterial infections, and detecting disease [254, 255, 256].

### 5.5 Biosensor Biorecognition

Traditional detection methodologies such as PCR use expensive reagents and complex instrumentation, are time‐consuming and laborious, and lack sensitivity [256, 257, 258]. In contrast, next‐generation nucleic acid biosensors (NABs) based on CRISPR‐Cas nucleases are robust, sensitive, easy to use, and rapid. Next‐generation NABs use immobilized nucleases as biosensor biorecognition elements to detect and quantify nucleic acid concentrations and specific sequences associated with disease, facilitating early diagnosis and positive patient outcomes [257]. For instance, the SARS‐CoV‐2 DNA Endonuclease‐Targeted CRISPR Trans Reporter (DETECTR) is commercially available (https://mammoth.bio/diagnostics/). In this kit, specific SARS‐CoV‐2 genome sequences are amplified and bind to the Cas12 sgRNA [259]. Upon binding, the Cas12 nuclease cleaves reporter molecules that can either be detected by lateral flow or fluorescence. Additionally, Cas9 nuclease immobilized on a graphene field‐effect transistor (CRISPR‐Chip) can detect the presence of specific mutations within whole HEK293T genomes [257]. This system was developed and commercialized by CRISPR QC (https://crisprqc.com/technology/) to detect mutated exons, commonly associated with Duchenne muscular dystrophy in clinical DNA samples. While these CRISPR‐based diagnostics have seen success, their application is severely hindered by the need for different sgRNAs to detect different mutations and pathogens. However, the DETECTR system has demonstrated the ability to detect different SARS‐CoV‐2 genes, which suggests that future innovations might allow the simultaneous detection of various mutations and pathogens. This could potentially be realised by multiple sgRNAs, different lateral flow systems, or various fluorescent reporter molecules.

### 5.6 Nuclease Secretion

To prevent IB sequestration, nucleases can be expressed in a soluble form using signal peptides that direct them to the extracellular environment or periplasm. Thus, a major advantage of this approach is that toxic proteins can be expressed without killing the heterologous host. Additional advantages of this approach over IBs and refolding include easier and faster purification, correctly folded proteins, and fewer endotoxins, which are released during the lysis of *E. coli* cells and are of regulatory concern [264, 275]. For example, the cytotoxicity of RNase A could be overcome by secretion to produce 100–300 mg/L compared to previous studies (0.2–8 mg/L), even when the tightly regulated T7 promoter and IB sequestration were used (Table 1) [183, 184, 185, 186]

While secretion provides several benefits, some of these will likely not always be realized. A major factor in the success of secretion is the correct signal peptide [195, 196]. The importance of an efficient signal peptide was demonstrated with SNase yields. By replacing the native SNase signal peptide with one that is better recognized and secreted by the heterologous expression host, SNase yields were improved from 1–2 to 115 mg/L (Table 1) [263, 264]. Additionally, by optimizing the cell density at which induction occurs and the strength of induction, SNase was efficiently matured and secreted. It is well‐known that overexpressing chaperones and secretion proteins can lead to inefficient secretion and protein maturation [276, 277]. Although a general consideration, the overexpression of nucleases aimed for secretion should be regulated to not overwhelm the cellular machinery. Alternatively, secretion can be improved by coexpressing a protein that induces cellular leakage. For example, the coexpression of a bacteriocin‐release protein and barnase (an RNase from *Bacillus amyloliquefaciens*) improved barnase secretion and allowed upscaling production, which was not previously possible (Table 1) [179].

Another important factor in the successful secretion of biologically active nuclease is whether disulfide bridges are required [264, 265]. While expression hosts such as *S. cerevisiae* can catalyze disulfide bridge formation, the reducing cytoplasm and plentiful reductases of *E. coli* hinder this process [166, 278]. One way to overcome this limitation is to fuse periplasmic signal peptides to nucleases. In the *E. coli* periplasm (Figure 1C), disulfide bridge formation is feasible due to the oxidative environment and local oxidoreductases [168, 278]. This approach was successfully illustrated with bovine pancreatic RNase A, which requires four disulfide bridges for activity [270]. Furthermore, 45–50 mg/L of protein was obtained, as opposed to 1 mg/L from conventional secretion by *Pichia pastoris* [271]. Similarly, the yields of several other nucleases have been increased by secretion (Table 1).

While secretion is a viable option, it has some drawbacks in addition to identifying the correct secretion signal. One drawback, particularly for traditional secretion, is that the initial cell harvesting step (Figure 1C, step C1) no longer has the benefit of a centrifuge‐based concentration step, and the full expression volume must be used to purify the nuclease of interest. Additionally, when higher yields of secreted nucleases are required, strong promoters or induction often lead to IB formation, which necessitates refolding [179, 202].

## 6 Nuclease Inhibitors

Although nuclease activity is desired in certain applications such as those discussed above, its activity can also disrupt or compromise the success of others. These applications' success relies either on complete inhibition of nucleases or on inhibiting nucleases after their role has been fulfilled [209, 210]. Nuclease inhibitors can either be protein‐based, such as *Anabaena* NuiA, or chemical agents that disrupt the structure and activity of nucleases [205]. Given the natural affinity between a nuclease and its inhibitor, immobilized inhibitors have been used as affinity chromatography ligands [278, 279, 280, 281, 282, 283]. Immunity protein 7 (Im7), the natural inhibitor of colicin E7 DNase (CE7), immobilized on Sulfo‐Link 6B agarose beads, could specifically purify CE7 [284]. The preparation of heterologous nucleases through inhibitor‐affinity chromatography provides superior purity and can exclude any other undesired nuclease activity, which is vital in certain applications. Inhibitor‐affinity chromatography has several advantages over traditional chromatography, including high selectivity, one‐step purification, the separation of inactive and active nuclease molecules, and rapid, scalable purification [285]. Generally, nucleotide analogs or derivatives that are also nuclease inhibitors are used as ligands to bind nucleases of interest with high specificity. For example, competitive inhibitors of SNase, cross‐linked to Sepharose resin, were used to selectively purify SNase in one step [285, 286]. Similarly, bovine and tobacco RNases were purified using Sepharose bound to 5′‐(4‐aminophenyl‐phosphoryl)‐uridine/guanosine‐2′(3′)‐phosphate, which are inhibitors of these RNases [287, 288, 289].

Research on protein‐based inhibitors as affinity chromatography ligands is limited, despite their application in nuclease purification being among the most efficient. The mechanism of nuclease inhibition can broadly be categorized into competitive, noncompetitive, and general inhibition. Competitive inhibition of nucleases is characterized by inhibitors that directly compete with nucleic acids to bind to the nucleases’ active sites [290, 291]. In contrast, noncompetitive inhibition occurs through structural changes from allosteric inhibitor binding that disrupt nuclease activity [292, 293]. General inhibition describes a broader class of inhibition mechanisms that differ from both competitive and noncompetitive inhibition. Examples include denaturation, metal cation chelation, and increased ionic strength.

### 6.1 Competitive Inhibition

Competitive nuclease inhibitors are important immune proteins that protect hosts from their nucleases by preventing intracellular nucleic acid degradation. Such immunity proteins have been documented in several species, including *Anabaena* spp., *B. amyloliquefaciens, S. aureus, E. coli, Homo sapiens, and M. musculus* [75, 120, 202, 205, 294]. While their major role is to prevent intracellular nuclease degradation and cell death, competitive nuclease inhibitors also play crucial roles in the regulation of apoptosis [295]. For example, without the inhibition of CAD and DFF40 by ICAD and DFF45, the nucleases would initiate apoptosis, resulting in unintentional cell death. Alternatively, nuclease inhibitors are used to protect hosts from non‐host nucleases. Nuclease EsaD (*S. aureus*) plays a key role in bacterial competition whereby sensitive strains, lacking the EsaD inhibitor, EsaG, are killed by EsaD. Generally, *S. aureus* strains encode two EsaG‐like proteins to protect themselves from EsaD and ensure propagation and proliferation. Similarly, anti‐CRISPR (Acr) proteins have evolved in bacteriophages in response to evolutionary pressure from bacterial and archaeal CRISPR‐Cas immunity systems [90, 140, 142]. For example, Acr proteins that directly bind to Cas nucleases or prevent binding of the sgRNA have been described in *Neisseria meningitidis* and *P. aeruginosa* [140, 142].

### 6.2 Noncompetitive Inhibition

Noncompetitive nuclease inhibitors have been found to have similar roles to competitive inhibitors. For example, an Acr protein (AcrF1) binds to an allosteric protein subunit outside of the active site of Cas7f [296]. The inhibition of Cas7f by AcrF1 is caused by the inability of Cas7f to fold over and relocate the crRNA to the nuclease domain [297]. Similar to competitive inhibitors, non‐competitive inhibitors are also crucial during apoptosis. An inhibitor of *Aspergillus oryzae*’s nsNuclease O is non‐competitively inhibited through a mutual depletion system [298]. This inhibitor is crucial during the late growth phase and prevents cell death before apoptosis and autolysis. Interestingly, an inhibitor of *Neurospora crassa* endo‐exonuclease is crucial to regulate its ssDNAse, dsDNAse, and RNAse activity [299]. Depending on the desired activity, the inhibitor can either bind to an allosteric site or the active site, thereby eliciting both noncompetitive (ssDNase and ssRNase) and competitive inhibition (dsDNase). Noncompetitive end‐product inhibition has also been observed for nucleases including *Bam*HI, poly(A)‐specific RNase, and RNase A [292, 293, 300]. The reason for this inhibition remains unknown, but it may be explained as a protective role. In this case, the inhibition prevents excessive build‐up of nuclease end products or prevents excessive nucleic acid degradation. While noncompetitive inhibition relies on a structural change, general inhibition relies on several inhibitory mechanisms, including structural changes, changes to electrostatic interactions, and cofactor chelation.

### 6.3 General Inhibition

Outside of competitive and noncompetitive inhibition, several other inhibitory agents of nuclease activity exist. Since the activity of enzymes such as nucleases is heavily dependent on their structure, any disruptions to this structure will result in inhibition. While several chemical and physicochemical agents capable of nuclease denaturation exist, only a few will be discussed.

Reductants such as *β‐*mercaptoethanol and dithiothreitol can inhibit disulfide bridge‐containing nucleases by reduction [301]. Without these disulfide bridges, the tertiary structure becomes disrupted, which inhibits the activity of nucleases such as *Ustilago maydis* nuclease α [302, 303].

Similarly, at high enough concentrations, denaturants such as urea can inhibit the activity of all nucleases. While the mechanism of denaturation is not fully understood, the denaturation of the protein structure results in the inhibition of nuclease activity [304]. It is hypothesized that urea‐induced denaturation is a result of disrupted hydrogen bonds and hydrophobic interactions.

While urea is hypothesized to disrupt interactions between amino acids in nucleases, monovalent cations can inhibit nucleases by disrupting electrostatic interactions between the nuclease and its substrate [305, 306]. Additionally, monovalent cations such as sodium and potassium can shield the negative charge of nucleic acids or reduce the helicity of DNA, which inhibits nuclease activity [279].

Lastly, metal chelators such as EDTA (ethylenediaminetetraacetic acid) inhibit nucleases by chelating metal cation cofactors [298]. This inhibition results from structural disruption and cofactor removal. An example is the removal of Zn^2+^ ions from nuclease P1 (*Penicillium citratum*) by EDTA treatment, disrupting its active conformation and resulting in diminished activity [280]. Additionally, metal cation cofactors have also been implicated in substrate binding. For example, mutagenesis showed that a second Mg^2+^ ion is essential for substrate binding in bacteriophage T4 RNase H [281]. Therefore, cofactor removal can disrupt the structure and binding capacity of nucleases to nucleic acids.

Taken together, nucleases can be inhibited through various mechanisms and agents. Nuclease inhibition is extremely important for the survival and propagation of species. Additionally, these inhibitors play a crucial role in the expression of otherwise cytotoxic nucleases in heterologous hosts. Nuclease inhibitors have also been used in various niche applications where the inhibition of nucleases is crucial for successful outcomes.

## 7 Nucleic Acid Protection in Cell‐Free Protein Synthesis

Nuclease inhibitors have a wide range of applications in various in vitro experimental research and industrial activities. Given their usefulness, several protein‐based nuclease inhibitors, most of which target RNases, have been commercialized. Generally, nuclease inhibitors, protein‐based or chemical agents, are used in two main applications, including nucleic acid protection and affinity chromatography.

In nucleic acid protection applications, inhibitors can either be added from the start to completely prevent nucleic acid degradation or after nuclease action. One such application is cell‐free protein synthesis (CFPS), which relies on successful in vitro transcription (IVT) and translation [282]. Partially purified lysates containing cellular gene‐expression components are used for CFPS. However, generally, CFPS yields are extremely low because of residual endogenous ribonucleases. Therefore, ribonuclease inhibitors are an asset during IVT and translation by inhibiting endogenous RNases that would otherwise degrade the mRNA templates [210]. The *E. coli* RecBCD nuclease domain is another enzyme that contributes to low yields by degrading DNA templates. However, adding a bacteriophage *λ* inhibitor of *E. coli* RecBCD exonuclease, GamS, increased yields of aglycosylated antibodies and antibody fragments with high efficiency [283]. Additionally, CFPS also benefits from the exogenous nuclease‐based degradation of endogenous nucleic acids followed by the addition of nuclease inhibitors [307]. This approach increases CFPS yields by allowing preferential IVT of exogenous DNA.

Furthermore, the success of total and miRNA isolation for subsequent real‐time PCR is heavily dependent on the quantity and quality of the RNA [308]. It is of utmost importance to extract high quantities of RNA for the detection of several diseases. In these circumstances, the use of RNase inhibitors has decreased false positives, increased the limit of detection, and extended the storage times of samples obtained from patients potentially suffering from SARS‐CoV‐2 and preeclampsia [308, 309]. Furthermore, the miRNA could be used to alter the miRNAome of postpartum preeclampsia patients, which would ameliorate the condition in suffering patients.

Nuclease inhibitors are also commonly used in laboratories during nucleic acid isolation, purification, and storage [310]. For example, the final step of manual DNA preparation includes resuspending the DNA pellet in a Tris‐HCl buffer containing EDTA to protect the extracted DNA from metal‐cation‐dependent nucleases [210]. Similarly, NaCl can be used to inhibit nucleases during RNA or DNA extractions [311]. Lastly, nuclease inhibitors are used in agarose gel electrophoresis buffers to prevent the degradation of nucleic acids [209, 210]. These inhibitors are often used in molecular extraction kits provided by Thermo Fisher Scientific, NEB, and Promega to prevent nucleic acid degradation. The addition of nuclease inhibitors in these applications further enables other methods such as cloning, PCR, sequencing, and host transformation, which serve as the backbone for research and biotechnology.

## 8 Conclusion

The study of nucleases and their diverse applications continues to reveal the immense potential of these enzymes and their inhibitors across multiple industries. Applications of nucleases stretch from therapeutic interventions to genetic engineering and diagnostics. Moreover, advancements in nuclease discovery, expression, and characterization have shown promise in enhancing laboratory and industrial applications such as vaccine manufacturing, nucleic acid and heterologous protein isolations, and genetic engineering. Despite these significant developments, several challenges, such as their overexpression and stability, remain. The literature reveals a growing body of work aimed at addressing these challenges; however, a more focused approach is required to facilitate the commercial viability and scalability of nuclease‐based technologies, especially in South Africa. Moreover, additional focus should be placed on discovering and characterizing nuclease inhibitors, particularly nsNuclease inhibitors, of which none have been commercialized to date.

## Author Contributions

**Wian Vermeulen**: investigation, writing – original draft. **Anton Du Preez van Staden**: conceptualization, methodology. **Leon M.T. Dicks**: conceptualization, writing – review and editing, validation, project administration, supervision.

## Conflicts of Interest

The authors declare no conflicts of interest.

## Data Availability

Data sharing is not applicable to this article as no datasets were generated or analyzed during the current study.
